# 4-[(2-Hy­droxy­benz­yl)amino]­pyridinium nitrate

**DOI:** 10.1107/S1600536812031352

**Published:** 2012-07-18

**Authors:** Shan Gao, Seik Weng Ng

**Affiliations:** a Key Laboratory of Functional Inorganic Materials Chemistry, Ministry of Education, Heilongjiang University, Harbin 150080, People’s Republic of China; b Department of Chemistry, University of Malaya, 50603 Kuala Lumpur, Malaysia; c Chemistry Department, Faculty of Science, King Abdulaziz University, PO Box 80203, Jeddah, Saudi Arabia

## Abstract

The planes of the aromatic rings in the cation of the title salt, C_12_H_13_N_2_O^+^·NO_3_^−^, are twisted along the –CH_2_—NH– single bond by 75.3 (1)°. In the crystal, the phenol O, amine N and pyridinium N atoms are hydrogen-bond donors to the O atoms of the nitrate counter-ions. These hydrogen bonds lead to the formation of a layer in the crystal.

## Related literature

For 2-[(pyridin-2-yl­amino)­meth­yl]phenol, see: Gao & Ng (2012 ▶). For 2-[(pyridin-3-yl­amino)­meth­yl]phenol, see: Xu *et al.* (2011 ▶).
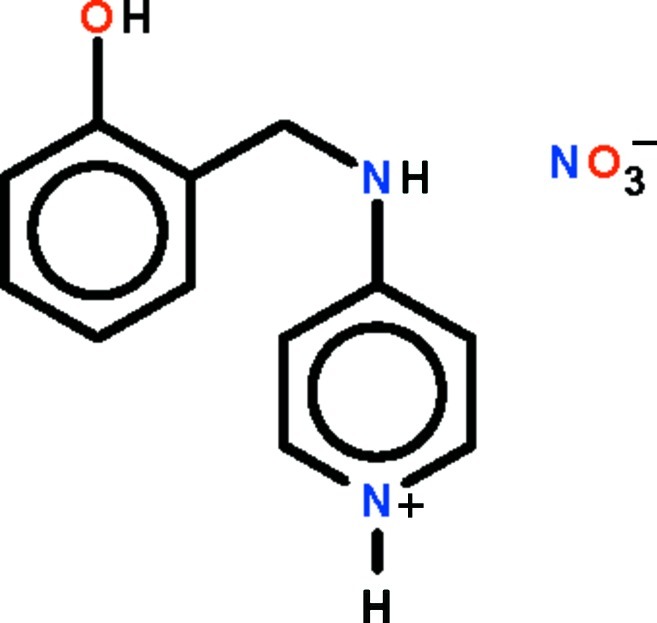


## Experimental

### 

#### Crystal data

C_12_H_13_N_2_O^+^·NO_3_^−^
*M* *_r_* = 263.25
Monoclinic, 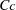

*a* = 13.611 (4) Å
*b* = 12.687 (3) Å
*c* = 10.030 (2) Å
β = 132.694 (12)°
*V* = 1273.0 (5) Å^3^
*Z* = 4
Mo *K*α radiation
μ = 0.11 mm^−1^
*T* = 295 K
0.24 × 0.21 × 0.21 mm

#### Data collection

Rigaku R-AXIS RAPID IP diffractometer
Absorption correction: multi-scan (*ABSCOR*; Higashi, 1995 ▶) *T*_min_ = 0.975, *T*_max_ = 0.978
6077 measured reflections
1458 independent reflections
1176 reflections with *I* > 2σ(*I*)
*R*_int_ = 0.041

#### Refinement

*R*[*F* ^2^ > 2σ(*F* ^2^)] = 0.043
*wR*(*F* ^2^) = 0.116
*S* = 1.08
1458 reflections
184 parameters
5 restraints
H atoms treated by a mixture of independent and constrained refinement
Δρ_max_ = 0.20 e Å^−3^
Δρ_min_ = −0.17 e Å^−3^

### 

Data collection: *RAPID-AUTO* (Rigaku, 1998 ▶); cell refinement: *RAPID-AUTO*; data reduction: *CrystalClear* (Rigaku/MSC, 2002 ▶); program(s) used to solve structure: *SHELXS97* (Sheldrick, 2008 ▶); program(s) used to refine structure: *SHELXL97* (Sheldrick, 2008 ▶); molecular graphics: *X-SEED* (Barbour, 2001 ▶); software used to prepare material for publication: *publCIF* (Westrip, 2010 ▶).

## Supplementary Material

Crystal structure: contains datablock(s) global, I. DOI: 10.1107/S1600536812031352/xu5582sup1.cif

Structure factors: contains datablock(s) I. DOI: 10.1107/S1600536812031352/xu5582Isup2.hkl

Supplementary material file. DOI: 10.1107/S1600536812031352/xu5582Isup3.cml

Additional supplementary materials:  crystallographic information; 3D view; checkCIF report

## Figures and Tables

**Table 1:** Hydrogen-bond geometry (Å, °)

*D*—H⋯*A*	*D*—H	H⋯*A*	*D*⋯*A*	*D*—H⋯*A*
O1—H1⋯O2	0.84 (2)	1.97 (2)	2.800 (3)	169 (4)
N1—H3⋯O2^i^	0.88 (3)	2.33 (2)	3.017 (3)	134 (2)
N1—H3⋯O3^i^	0.88 (3)	2.00 (3)	2.860 (4)	165 (3)
N2—H2⋯O3^ii^	0.88 (1)	2.36 (2)	3.089 (3)	141 (3)
N2—H2⋯O4^ii^	0.88 (1)	2.18 (2)	3.027 (3)	162 (3)

Symmetry codes: (i) 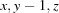; (ii) 
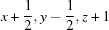
.

**Fig. 1.**
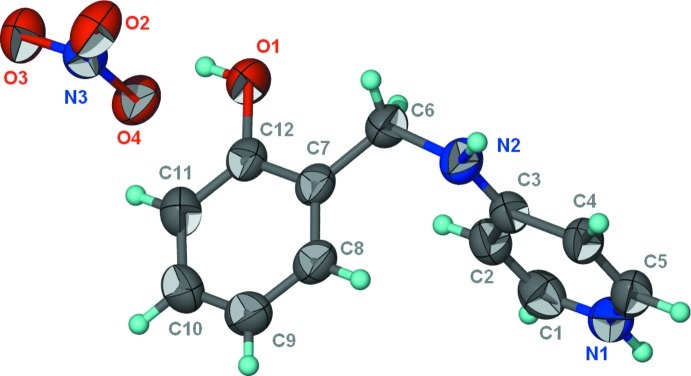
Thermal ellipsoid plot (Barbour, 2001) of C12H13N2O.NO3 at the 50% probability level; hydrogen atoms are drawn as spheres of arbitrary radius.

**Fig. 2.**
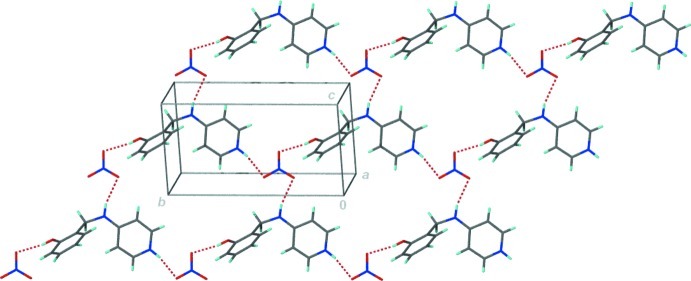
Hydrogen-bonded network motif.

## supplementary crystallographic information

### Comment

Salicylaldehyde condenses with aromatic amines to yield Schiff bases, which serve as chelating ligands to a plethora of metal systems. These Schiff bases can be readily reduce to the corresponding secondary amines, which can also function as chelating ligands. Curiously, there are only few 2-(arylamino)methylphenols compared with the plethora of Schiff bases in the chemical literature. Among the aminopyridine derivatives, only the crystal structures of 2-((pyridin-2-ylamino)methyl)phenol (Gao & Ng, 2012) and 2-((pyridin-3-ylamino)methyl)phenol (Xu *et al.*, 2011) analogs have been reported. The 2-((pyridin-4-ylamino)methyl)phenol analog is now authenticated as its nitrate salt (Scheme I).

The two aromatic rings of the reduced Schiff-base salt, C_12_H_13_N_2_O^.^NO_3_, are twisted along the –CH_2_–NH– single-bond by 75.3 (1) ° (Fig. 1). The hydroxy O, amino N and pyridinium N atoms are each a hydrogen-bond donor to an O atom of the nitrate counterion. These hydrogen bonds lead to the formation of a layer parallel to [1 0 1] (Fig. 2, Table 1).

### Experimental

A solution of 4-aminopyridine (1 mmol) and salicylaldehyde (1 mmol) in toluene (50 ml) was heated for 10 h. The solvent was removed under vacuum, and the residue was reduced in absolutem ethanol by sodium borohydride. Light yellow crystals were obtained by recrystallization from methanol to which several drops of nitric acid were added.

### Refinement

Carbon-bound H-atoms were placed in calculated positions (C–H 0.93 Å) and were included in the refinement in the riding model approximation, with *U*(H) set to 1.2*U*(C). The amino and hydroxy H-atoms were located in a difference Fourier map, and were refined with distance restraints N–H 0.88±0.01 Å, O–H 0.84±0.01 Å; their temperature factors were refined.

In the absence of heavy scatters, 1320 Friedel pairs were merged.

### Crystal data

**Table:**

C_12_H_13_N_2_O^+^·NO_3_^−^	*Z* = 4
*M**_r_* = 263.25	*F*(000) = 552
Monoclinic, *C**c*	*D*_x_ = 1.374 Mg m^−^^3^
Hall symbol: C -2yc	Mo *K*α radiation, λ = 0.71073 Å
*a* = 13.611 (4) Å	µ = 0.11 mm^−^^1^
*b* = 12.687 (3) Å	*T* = 295 K
*c* = 10.030 (2) Å	Prism, faint yellow
β = 132.694 (12)°	0.24 × 0.21 × 0.21 mm
*V* = 1273.0 (5) Å^3^	

### Data collection

**Table:**

Rigaku R-AXIS RAPID IP diffractometer	1458 independent reflections
Radiation source: fine-focus sealed tube	1176 reflections with *I* > 2σ(*I*)
Graphite monochromator	*R*_int_ = 0.041
ω scan	θ_max_ = 27.5°, θ_min_ = 3.2°
Absorption correction: multi-scan (*ABSCOR*; Higashi, 1995)	*h* = −17→17
*T*_min_ = 0.975, *T*_max_ = 0.978	*k* = −16→16
6077 measured reflections	*l* = −13→13

### Refinement

**Table:**

Refinement on *F*^2^	Primary atom site location: structure-invariant direct methods
Least-squares matrix: full	Secondary atom site location: difference Fourier map
*R*[*F*^2^ > 2σ(*F*^2^)] = 0.043	Hydrogen site location: inferred from neighbouring sites
*wR*(*F*^2^) = 0.116	H atoms treated by a mixture of independent and constrained refinement
*S* = 1.08	*w* = 1/[σ^2^(*F*_o_^2^) + (0.0753*P*)^2^] where *P* = (*F*_o_^2^ + 2*F*_c_^2^)/3
1458 reflections	(Δ/σ)_max_ = 0.001
184 parameters	Δρ_max_ = 0.20 e Å^−^^3^
5 restraints	Δρ_min_ = −0.17 e Å^−^^3^

### Special details

**Table:**

Geometry. All e.s.d.'s (except the e.s.d. in the dihedral angle between two l.s. planes) are estimated using the full covariance matrix. The cell e.s.d.'s are taken into account individually in the estimation of e.s.d.'s in distances, angles and torsion angles; correlations between e.s.d.'s in cell parameters are only used when they are defined by crystal symmetry. An approximate (isotropic) treatment of cell e.s.d.'s is used for estimating e.s.d.'s involving l.s. planes.

### Fractional atomic coordinates and isotropic or equivalent isotropic displacement parameters (Å^2^)

**Table:**

	*x*	*y*	*z*	*U*_iso_*/*U*_eq_	
O1	0.5006 (2)	0.17761 (17)	0.5005 (3)	0.0795 (6)	
O2	0.4295 (3)	0.38568 (15)	0.3747 (3)	0.0882 (7)	
O3	0.3096 (2)	0.47126 (17)	0.1249 (3)	0.0817 (6)	
O4	0.3075 (2)	0.30247 (17)	0.1215 (3)	0.0816 (6)	
N1	0.4372 (3)	−0.37774 (19)	0.4116 (4)	0.0740 (6)	
N2	0.6073 (2)	−0.11750 (18)	0.7349 (3)	0.0692 (6)	
N3	0.3475 (2)	0.38556 (18)	0.2053 (3)	0.0669 (6)	
C1	0.4016 (3)	−0.2829 (3)	0.3359 (4)	0.0739 (7)	
H1A	0.3378	−0.2776	0.2101	0.089*	
C2	0.4552 (3)	−0.1938 (2)	0.4358 (4)	0.0660 (6)	
H2A	0.4293	−0.1284	0.3792	0.079*	
C3	0.5510 (2)	−0.2009 (2)	0.6271 (4)	0.0588 (6)	
C4	0.5857 (3)	−0.3036 (2)	0.7024 (4)	0.0641 (6)	
H4	0.6484	−0.3128	0.8276	0.077*	
C5	0.5270 (3)	−0.3885 (2)	0.5910 (4)	0.0720 (7)	
H5	0.5501	−0.4557	0.6411	0.086*	
C6	0.5830 (3)	−0.0101 (2)	0.6719 (4)	0.0708 (7)	
H6A	0.5908	−0.0060	0.5828	0.085*	
H6B	0.6522	0.0343	0.7734	0.085*	
C7	0.4489 (2)	0.03351 (19)	0.5886 (3)	0.0569 (5)	
C8	0.3625 (3)	−0.0166 (2)	0.5960 (4)	0.0650 (6)	
H8	0.3879	−0.0800	0.6584	0.078*	
C9	0.2390 (3)	0.0260 (3)	0.5123 (5)	0.0784 (8)	
H9	0.1825	−0.0078	0.5201	0.094*	
C10	0.2006 (3)	0.1180 (3)	0.4180 (6)	0.0834 (9)	
H10	0.1164	0.1456	0.3584	0.100*	
C11	0.2847 (3)	0.1707 (2)	0.4096 (4)	0.0742 (7)	
H11	0.2578	0.2336	0.3456	0.089*	
C12	0.4100 (3)	0.12908 (19)	0.4978 (3)	0.0607 (6)	
H1	0.477 (4)	0.2368 (14)	0.450 (4)	0.082 (9)*	
H2	0.670 (3)	−0.126 (3)	0.8524 (16)	0.081 (10)*	
H3	0.400 (3)	−0.4320 (18)	0.337 (4)	0.081 (10)*	

### Atomic displacement parameters (Å^2^)

**Table:**

	*U*^11^	*U*^22^	*U*^33^	*U*^12^	*U*^13^	*U*^23^
O1	0.0804 (12)	0.0576 (10)	0.1069 (16)	0.0045 (9)	0.0660 (12)	0.0162 (10)
O2	0.1108 (18)	0.0634 (12)	0.0670 (14)	−0.0060 (11)	0.0509 (14)	−0.0011 (9)
O3	0.0915 (14)	0.0660 (12)	0.0782 (13)	0.0112 (10)	0.0539 (12)	0.0097 (10)
O4	0.0855 (13)	0.0665 (12)	0.0805 (13)	−0.0092 (10)	0.0514 (12)	−0.0138 (9)
N1	0.0745 (14)	0.0683 (15)	0.0895 (17)	−0.0068 (12)	0.0597 (14)	−0.0155 (13)
N2	0.0629 (13)	0.0570 (13)	0.0657 (14)	0.0038 (9)	0.0349 (12)	0.0069 (10)
N3	0.0683 (12)	0.0654 (15)	0.0713 (14)	0.0001 (10)	0.0490 (12)	−0.0010 (10)
C1	0.0661 (15)	0.0879 (19)	0.0658 (15)	0.0049 (14)	0.0440 (13)	−0.0032 (14)
C2	0.0633 (14)	0.0672 (15)	0.0679 (15)	0.0097 (12)	0.0446 (13)	0.0088 (12)
C3	0.0562 (12)	0.0546 (13)	0.0682 (15)	0.0055 (10)	0.0432 (13)	0.0058 (10)
C4	0.0664 (15)	0.0564 (14)	0.0701 (15)	0.0080 (11)	0.0465 (13)	0.0094 (11)
C5	0.0798 (17)	0.0559 (15)	0.095 (2)	0.0059 (12)	0.0648 (17)	0.0061 (13)
C6	0.0637 (13)	0.0531 (14)	0.0834 (17)	−0.0003 (11)	0.0450 (13)	0.0036 (12)
C7	0.0619 (13)	0.0460 (11)	0.0593 (12)	−0.0046 (9)	0.0397 (11)	−0.0073 (9)
C8	0.0780 (15)	0.0521 (12)	0.0761 (15)	−0.0087 (12)	0.0566 (13)	−0.0094 (11)
C9	0.0841 (18)	0.0707 (18)	0.108 (2)	−0.0118 (14)	0.0759 (19)	−0.0190 (16)
C10	0.0751 (17)	0.075 (2)	0.112 (2)	0.0063 (14)	0.0680 (19)	−0.0095 (17)
C11	0.0767 (16)	0.0611 (15)	0.0868 (19)	0.0102 (12)	0.0562 (16)	0.0047 (12)
C12	0.0665 (13)	0.0504 (13)	0.0675 (14)	−0.0021 (11)	0.0463 (12)	−0.0055 (10)

### Geometric parameters (Å, º)

**Table:**

O1—C12	1.362 (3)	C4—C5	1.354 (4)
O1—H1	0.837 (10)	C4—H4	0.9300
O2—N3	1.250 (3)	C5—H5	0.9300
O3—N3	1.238 (3)	C6—C7	1.505 (4)
O4—N3	1.222 (3)	C6—H6A	0.9700
N1—C1	1.327 (4)	C6—H6B	0.9700
N1—C5	1.330 (4)	C7—C8	1.383 (4)
N1—H3	0.882 (10)	C7—C12	1.388 (3)
N2—C3	1.324 (4)	C8—C9	1.381 (4)
N2—C6	1.443 (3)	C8—H8	0.9300
N2—H2	0.874 (10)	C9—C10	1.364 (5)
C1—C2	1.350 (4)	C9—H9	0.9300
C1—H1A	0.9300	C10—C11	1.376 (5)
C2—C3	1.413 (4)	C10—H10	0.9300
C2—H2A	0.9300	C11—C12	1.387 (4)
C3—C4	1.418 (3)	C11—H11	0.9300
			
C12—O1—H1	115 (3)	N2—C6—C7	115.0 (2)
C1—N1—C5	120.7 (3)	N2—C6—H6A	108.5
C1—N1—H3	116 (2)	C7—C6—H6A	108.5
C5—N1—H3	123 (2)	N2—C6—H6B	108.5
C3—N2—C6	124.3 (2)	C7—C6—H6B	108.5
C3—N2—H2	120 (2)	H6A—C6—H6B	107.5
C6—N2—H2	116 (2)	C8—C7—C12	118.5 (2)
O4—N3—O3	121.0 (2)	C8—C7—C6	123.8 (2)
O4—N3—O2	120.5 (2)	C12—C7—C6	117.7 (2)
O3—N3—O2	118.5 (2)	C9—C8—C7	121.1 (3)
N1—C1—C2	122.0 (3)	C9—C8—H8	119.5
N1—C1—H1A	119.0	C7—C8—H8	119.5
C2—C1—H1A	119.0	C10—C9—C8	119.5 (3)
C1—C2—C3	119.5 (3)	C10—C9—H9	120.2
C1—C2—H2A	120.3	C8—C9—H9	120.2
C3—C2—H2A	120.3	C9—C10—C11	121.0 (3)
N2—C3—C2	123.3 (2)	C9—C10—H10	119.5
N2—C3—C4	120.1 (2)	C11—C10—H10	119.5
C2—C3—C4	116.7 (2)	C10—C11—C12	119.3 (3)
C5—C4—C3	119.6 (3)	C10—C11—H11	120.3
C5—C4—H4	120.2	C12—C11—H11	120.3
C3—C4—H4	120.2	O1—C12—C11	123.0 (2)
N1—C5—C4	121.5 (3)	O1—C12—C7	116.4 (2)
N1—C5—H5	119.3	C11—C12—C7	120.6 (2)
C4—C5—H5	119.3		
			
C5—N1—C1—C2	0.8 (4)	N2—C6—C7—C12	−169.2 (2)
N1—C1—C2—C3	−0.8 (4)	C12—C7—C8—C9	1.3 (4)
C6—N2—C3—C2	−2.9 (4)	C6—C7—C8—C9	−178.1 (3)
C6—N2—C3—C4	177.4 (2)	C7—C8—C9—C10	1.2 (4)
C1—C2—C3—N2	−179.3 (3)	C8—C9—C10—C11	−2.0 (5)
C1—C2—C3—C4	0.5 (3)	C9—C10—C11—C12	0.3 (5)
N2—C3—C4—C5	179.6 (3)	C10—C11—C12—O1	−178.2 (3)
C2—C3—C4—C5	−0.1 (3)	C10—C11—C12—C7	2.1 (4)
C1—N1—C5—C4	−0.4 (4)	C8—C7—C12—O1	177.4 (2)
C3—C4—C5—N1	0.1 (4)	C6—C7—C12—O1	−3.2 (3)
C3—N2—C6—C7	73.7 (4)	C8—C7—C12—C11	−2.9 (3)
N2—C6—C7—C8	10.2 (4)	C6—C7—C12—C11	176.5 (3)

### Hydrogen-bond geometry (Å, º)

**Table:**

*D*—H···*A*	*D*—H	H···*A*	*D*···*A*	*D*—H···*A*
O1—H1···O2	0.84 (2)	1.97 (2)	2.800 (3)	169 (4)
N1—H3···O2^i^	0.88 (3)	2.33 (2)	3.017 (3)	134 (2)
N1—H3···O3^i^	0.88 (3)	2.00 (3)	2.860 (4)	165 (3)
N2—H2···O3^ii^	0.88 (1)	2.36 (2)	3.089 (3)	141 (3)
N2—H2···O4^ii^	0.88 (1)	2.18 (2)	3.027 (3)	162 (3)

Symmetry codes: (i) *x*, *y*−1, *z*; (ii) *x*+1/2, *y*−1/2, *z*+1.
